# The Rubber Company and the American Dental Association

**Published:** 1866-11

**Authors:** 


### THE RUBBER COMPANY AND THE AMERICAN DENTAL ASSOCIATION.

*Company.*—"Gentlemen, we represent the interests of a company recently formed in Boston, that has purchased the rights of the Goodyear Patent Company so far as applied to dentistry, and the Cummings' patent, and wish to have a talk with you."

*Association.*—"We will listen to any proposition you wish to make."

*Co.*—"We propose that your profession, throughout the United States, pay us six dollars for the right on each piece of rubber made, this to include under the Goodyear Patent for six years longer, and the Cummings' patent for nine years more."

*Ass.*—"We will do no such thing. Your claims are unreasonable, and extortionate. 'Millions for defense; but not one cent for tribute.' We will fight you to the bitter end."

*Co.*—"Do not get excited. We are gentlemen all. But you see we are most amply protected by law. We have the Goodyear patent, fortified by a decision against Dr. Roberts, of New York, and another against Col. Toland, of Cincinnati; and the case in the Cummings' patent against Dr. Wetherbee, of Boston, agent of the Great Protective Union, was decided in our favor. So you see we are mailed from top to toe, and double clad besides."

*Ass.*—"Is that so?"

*Co.*—"Most assuredly; and we shall push our claims forthwith in the most energetic manner. But we will not be hard with you; we will let you off easy."

*Ass.*—"Very well. Suppose you say two dollars and a half for every piece having more than six teeth, and one dollar for each with six or less."

*Co.*—"We think those rates very cheap indeed, but we will accept them. And further, to convince you of our liberality, we will agree to take only half of what is due us for the first fourteen months; and, to make it still less objectionable at the start we will pay that all back after a while. We only want a little just now for the wife and children."

*Ass.*—"Well, that is very considerate. We will think of it. Will you protect us in using the rubber, and make all others pay?"

*Co.*—"We will certainly do so, and put it in the license."

*Ass.*—"Very well, we accede to your terms for the fifteen years, and will go on harmoniously."

Did not the Association act in the premises without constitutional or other authority? To be sure the Rubber Company kindly tell us the Association "had full power to act"—for the entire profession of course.

If the Profession submits to the extortionate and extraordinary demands agreed to by the Association it should only be when compelled to do so by a decision of the Supreme Court of the United States. OBSERVER.

